# Vaccination in Germany

**Published:** 1890-04-19

**Authors:** 


### VACCINATION IN GERMANY.

German subjects are vaccinated in the early months of life, again on attaining the age of twelve, and all males a third time on entering upon their term of compulsory service in the army. Vaccination is always done with calf-lymph; never from arm to arm. Ill consequences are stated to be unknown. Small-pox is likewise practically unknown. The law is strict; and resistance to it being perfectly useless is never met with.

